# ComPASS: A software tool for Care Preference Assessment of Satisfaction in Skilled Nursing

**DOI:** 10.1093/geroni/igaf122.4088

**Published:** 2025-12-31

**Authors:** Anthony Sterns, Katherine Abbott, Kimberly VanHaitsma, Charles De Vilmorin, Jeffry Moore

**Affiliations:** Miami University, Twinsburg, Ohio, United States; Miami University, Oxford, Ohio, United States; The Pennsylvania State University, University Park, Pennsylvania, United States; LifeLoop, LifeLoop, District of Columbia, United States; LifeLoop, LifeLoop, District of Columbia, United States

## Abstract

The results of the Compass project demonstrate both the feasibility and significant usefulness of digital resident preference assessments for enhancing person-centered care in nursing home communities. Using the scientifically validated Preferences for Everyday Living Inventory (PELI), Compass-21 was created, consisting of 21 targeted questions—16 aligned with MDS Section F and five additional high-priority preferences chosen by staff after reviewing the remaining 56 PELI questions. Focus groups and formative research with administrators and frontline staff revealed that existing paper-based and EHR systems were not dynamic enough and were poorly updated for sharing preference information, which hindered truly individualized care. Compass-21 enabled systematic, real-time documentation and sharing of residents’ preferences and satisfaction. In four nursing home communities, 72 eligible residents (with a Brief Inventory for Mental Status score of 12 or higher) completed assessments of their most important preferences and provided a satisfaction rating for how well each preference was met. The tool produced reports used in care planning meetings, facilitating targeted discussion and improvement efforts. A survey of the reports given to care staff (nursing, social work, activities), family, and residents showed that the reports were helpful (3.9/5.0), understandable (4.44/5.0), and useful (4.16/5.0). Statistical analyses examined the change in preference-congruent care by comparing baseline and 30-day follow-up using repeated measures ANOVA. These results showed positive increases in the number of important preferences that were met, providing a strong basis for expansion and wider adoption across communities.

